# A Comparative Analysis of National Health Strategies and Policy Documents in Selected Latin American and African Countries

**DOI:** 10.3390/ijerph23080986

**Published:** 2026-07-29

**Authors:** Andrés Mauricio Garcia-Sierra, Mahmud Sheku, Luz Adriana Urbina, Francisco Becerra-Posada, Carlos Espinal, Rodrigo DeAntonio, Diana Carolina Rivera-Pinzón

**Affiliations:** 1 EpiDriver, Building 230, 2nd Floor, Calle Jacinto Palacios, City of Knowledge (Ciudad del Saber), Clayton, Panama City 0843, Panama; mahmud.sheku@epidriver.com (M.S.); adriana.urbina@epidriver.com (L.A.U.); carolina.rivera@epidriver.com (D.C.R.-P.); 2 Department of Global Health, Robert Stempel College of Public Health & Social Work, Florida International University, 11200 SW 8th Street, AHC5, Miami, FL 33199, USA; fbecerra@fiu.edu (F.B.-P.); caespina@fiu.edu (C.E.); 3 Endpoints, Building 221, 4th Floor, Calle Jacinto Palacios, City of Knowledge (Ciudad del Saber), Clayton, Panama City 0843, Panama; rodrigo.deantonio@endpoints.site

**Keywords:** health equity, environmental health disparities, Latin America, Africa, vaccine access, social determinants of health, non-communicable diseases, infectious disease burden, climate-related health risks

## Abstract

**Highlights:**

**Public health relevance—How does this work relate to a public health issue?**

- Latin America and Africa face interconnected public health challenges driven by social inequities, environmental risks, and the dual burden of communicable and non-communicable diseases.
- Understanding shared and region-specific determinants of health across both regions is critical for designing equitable and effective health policies and interventions.

**Public health significance—Why is this work of significance to public health?**

- This study provides a comparative synthesis of national health strategies and epidemiological trends across multiple countries, highlighting structural drivers of health disparities.
- The analysis identifies systemic gaps in health care access, environmental health protections, and disease prevention affecting population health outcomes across both regions.

**Public health implications—What are the key implications or messages for practitioners, policy makers and/or researchers in public health?**

- Strengthening primary health care systems, improving domestic health financing, and addressing environmental determinants are essential for building resilient and equitable health systems.
- Cross-regional collaboration can support innovation, knowledge exchange, and sustainable strategies for addressing shared global health challenges.

**Abstract:**

Latin America and Africa face complex public health challenges shaped by social, environmental, and epidemiological factors. This study conducts a comparative analysis of publicly available national health strategies and national policy documents from 52 countries in Africa and 12 countries from Latin America, supplemented with data from the World Health Organization (WHO) and Pan American Health Organization (PAHO). The analysis focuses on five domains: social determinants of health, environmental health, infectious diseases, non-communicable diseases (NCDs), and immunization. This study examines how these health priorities are represented in national policy frameworks. Across the reviewed country sample, national policy documents tend to emphasize challenges related to health care access, the growing burden of NCDs, and environmental risks. In selected Latin American countries, migration-related pressures, health system fragmentation, and uneven vaccination coverage are primarily highlighted. In selected African countries, policy priorities tend to focus on communicable diseases, infrastructure limitations, and large zero-dose populations. The study underscores policy priorities and highlights opportunities for cross-regional learning. Strengthening primary health care, improving domestic health financing, and enhancing environmental health governance emerge as key areas for policy development. These findings support the value of comparative policy analysis in informing context-sensitive and equity-oriented health strategies.

## 1. Introduction

Public health systems worldwide face increasing pressure to respond to complex and evolving population health needs. Although Latin America and Africa differ in their political, economic, and historical contexts, both regions experience persistent health inequities, environmental challenges, and a dual burden of communicable and non-communicable diseases [1,2,3]. These shared challenges have driven countries to develop national health strategies aligned with global priorities such as Universal Health Coverage (UHC) and the Sustainable Development Goals (SDGs), while adapting these priorities to local health system contexts.

Social and environmental determinants remain major drivers of health outcomes across both regions. Inequities in income, education, housing, employment, access to health care, and environmental conditions contribute substantially to avoidable differences in disease burden and health service utilization, particularly among socially vulnerable populations [4,5]. Although these structural determinants are common to both regions, their manifestations differ according to demographic, institutional, and socioeconomic contexts.

Environmental risk factors continue to contribute substantially to population health. The World Health Organization (WHO) estimates that approximately one quarter of deaths in the WHO African Region are attributable to preventable environmental risks, including unsafe water, inadequate sanitation, household and ambient air pollution, and exposure to hazardous chemicals [6]. In Latin America, national health priorities increasingly address air and water pollution, deforestation, mining activities, and climate-related environmental threats, reflecting the growing recognition of environmental determinants within national public health agendas [7].

Non-communicable diseases (NCDs) have become the leading cause of mortality worldwide, accounting for approximately 74% of all deaths globally [8]. In Latin America and the Caribbean, NCDs are responsible for nearly 80% of all deaths, while their burden is increasing rapidly across Africa as countries undergo demographic and epidemiological transitions [9,10]. Cardiovascular diseases, cancer, diabetes, and chronic respiratory diseases increasingly challenge health systems that must simultaneously address persistent infectious diseases and expanding chronic care needs.

Despite substantial progress in disease prevention, infectious diseases remain major public health priorities in both regions. Many African countries continue to face high burdens of HIV, tuberculosis, and malaria, whereas Latin American countries tend to prioritize dengue, Chagas disease, tuberculosis, yellow fever, and other endemic infectious diseases [11]. Similarly, although immunization remains one of the most successful public health interventions, significant inequalities persist. According to WHO and UNICEF, an estimated 14.5 million children worldwide remained zero-dose in 2023, with a large proportion residing in African countries, while several Latin American countries continue efforts to recover routine vaccination coverage following disruptions associated with the COVID-19 pandemic [12].

National health strategies provide valuable insight into how governments prioritize investments to strengthen health system capacity and improve population health [13]. By defining priorities for disease prevention, health promotion, early diagnosis, treatment, workforce development, financing, and service delivery, these policies establish the strategic direction for achieving equitable access to quality care and advancing Universal Health Coverage (UHC) [14]. Although policy documents do not directly measure epidemiological outcomes or implementation, they reflect national priorities while incorporating guidance from international organizations such as the World Health Organization (WHO) and, in Latin America, the Pan American Health Organization (PAHO). Comparative analysis of these policy frameworks can therefore identify common priorities, regional differences, and opportunities for cross-regional learning.

Despite the growing number of national health strategies developed across Latin America and Africa, few studies have systematically compared how major public health priorities are framed across regions using a common analytical framework [15,16]. Most previous studies have focused on individual countries, specific diseases, or health system performance, with limited attention to cross-regional comparisons of national policy priorities [17]. Addressing this gap may provide valuable insights into shared challenges, region-specific priorities, and opportunities for mutual learning in health policy development.

This study conducts a structured comparative review of national health strategies and policy documents from selected countries in Latin America and Africa. The analysis focuses on five domains, social determinants of health, environmental health, infectious diseases, non-communicable diseases, and immunization, to examine how these priorities are articulated across national policy frameworks. Rather than evaluating health system performance or epidemiological outcomes, this review aims to identify shared policy priorities, contextual differences, and opportunities to inform context-sensitive and equity-oriented public health strategies.

## 2. Materials and Methods

This study employed a structured comparative document review of publicly available national health strategy documents and supporting literature to examine how major public health priorities are framed across selected Latin American and African countries. The review focused on five predefined analytical domains: infectious diseases, non-communicable diseases (NCDs), immunization, social determinants of health, and environmental health. These are the same analytical domains introduced in the Introduction and were used consistently throughout the review.

### 2.1. Study Design and Search Strategy

Relevant sources were identified through systematic searches of national health sector strategic plans, policy briefs, and official health reports published by ministries of health and regional agencies. Where available, official URLs and database access dates were recorded to facilitate reproducibility and verification of publicly accessible data sources. The search was limited to documents published between 2015 and 2023 to ensure contemporary policy relevance. The timeframe (2015–2023) was selected to capture the most recent cycle of national health strategies, which typically spans 5–10 years. This period reflects contemporary policy priorities, including post-SDG alignment and pre- and post-COVID-19 health system responses. However, earlier policy developments are not captured and may limit longitudinal interpretation.

### 2.2. Country Selection and Inclusion Criteria

Country inclusion was based primarily on document availability and accessibility. A convenience sampling approach was used to identify countries with publicly available national health strategy documents suitable for review. Subsequently, efforts were made to include countries from different subregions of Latin America and Africa and to capture variation in health system organization and epidemiological profiles. Consequently, the sample should be understood as illustrative rather than representative of either region.

**Figure 1:**
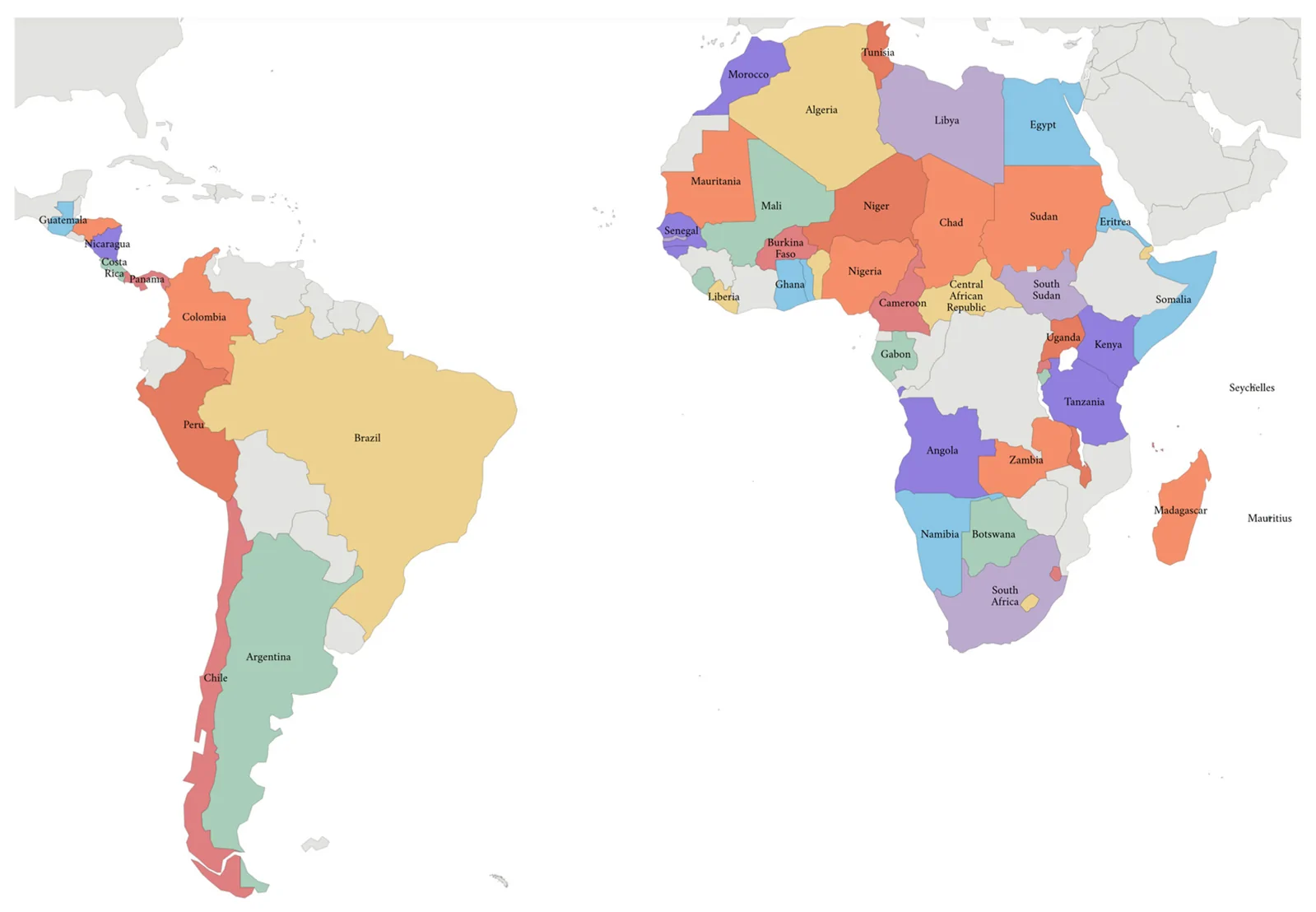
Countries included in the comparative review across Latin America and Africa.

For Latin America, twelve countries met these criteria: Argentina, Brazil, Chile, Colombia, Costa Rica, El Salvador, Guatemala, Honduras, Nicaragua, Panama, Peru, and the Dominican Republic (Figure 1). While this sample does not include all 20 Latin American countries, it captures a range of socioeconomic and health system contexts.

Similarly, 52 African countries were included based on document availability and to ensure representation across the continent’s major subregions. The African sample comprised 16 countries from West Africa (Benin, Burkina Faso, Cape Verde, The Gambia, Ghana, Guinea, Guinea-Bissau, Côte d’Ivoire, Liberia, Mali, Mauritania, Niger, Nigeria, Senegal, Sierra Leone, and Togo); 15 countries from East Africa (Burundi, Comoros, Djibouti, Eritrea, Ethiopia, Kenya, Madagascar, Malawi, Mauritius, Rwanda, Seychelles, Somalia, South Sudan, Tanzania, and Uganda); 7 countries from North Africa (Algeria, Egypt, Libya, Morocco, Sudan, Tunisia, and Western Sahara); 5 countries from Southern Africa (Botswana, Lesotho, Namibia, South Africa, and Eswatini); and 9 countries from Central Africa (Angola, Chad, Equatorial Guinea, Gabon, Cameroon, Central African Republic, Democratic Republic of the Congo, Republic of the Congo, and São Tomé and Príncipe). Together, these countries represent a broad range of socioeconomic, demographic, and health system contexts across the African continent (Figure 1). We acknowledge that the larger number of African countries compared to Latin American countries reflects differences in document availability and introduces an imbalance that may affect comparability. This limitation is addressed in the Section 4. Accordingly, the study should not be interpreted as providing a statistically representative comparison between regions, but rather as a comparative policy review of selected countries reflecting diverse health system contexts and policy priorities.

### 2.3. Analytical Framework

**Figure 2:**
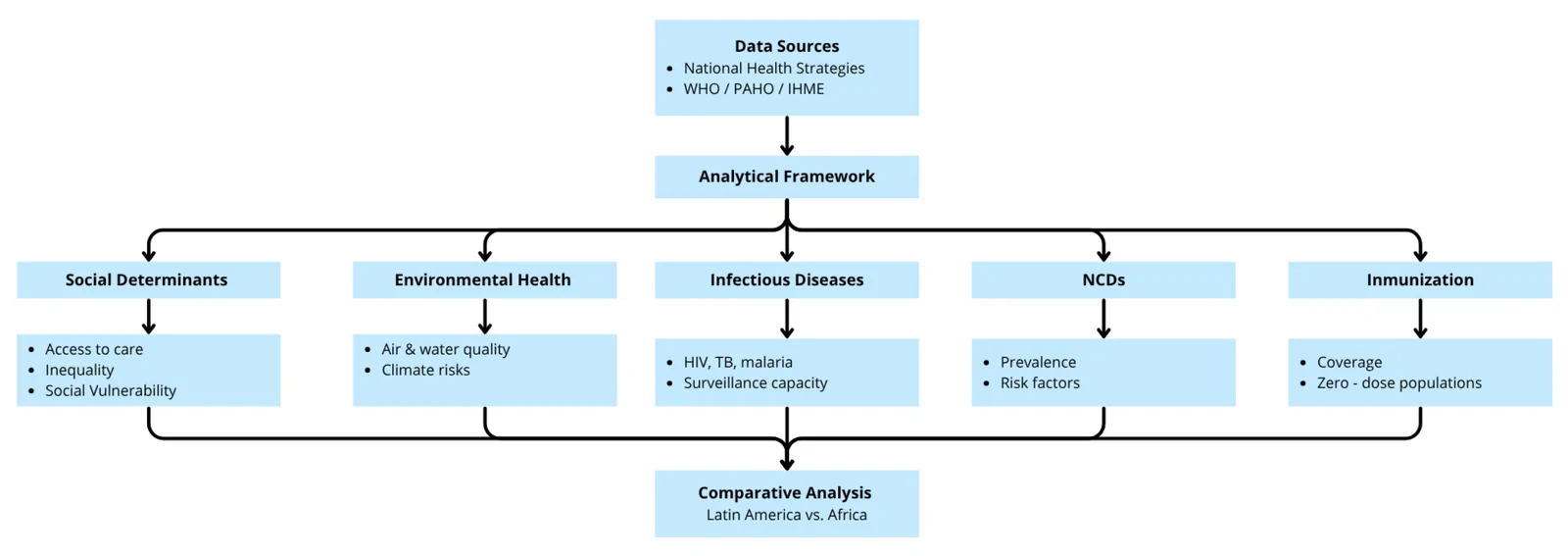
Analytical framework for comparative assessment of public health priorities in selected countries in Latin America and Africa. Source: Own elaboration. National health strategy and policy documents served as the primary data source for the structured comparative document review. WHO (Geneva, Switzerland), PAHO (Washington, DC, USA), IHME (Seattle, WA, USA), and other international sources were used exclusively to provide contextual background and inform the analytical framework; they were not included as primary analytical data for the comparison of national policy documents.

A deductive analytical framework was developed a priori by the authors to facilitate consistent cross-country comparison. The five analytical domains were selected because they represent key dimensions of contemporary public health policy and collectively capture structural, environmental, epidemiological, and preventive priorities primarily addressed in national health strategies. Their use provided a consistent framework for comparing policy priorities across countries with different health system contexts. The analytical framework guiding this study is presented in Figure 2. It organizes health priorities into five domains: (1) social determinants of health, (2) environmental health, (3) infectious diseases, (4) non-communicable diseases, and (5) immunization. Each domain includes key indicators extracted from policy documents and global data sources, allowing for structured comparison across countries and regions.

### 2.4. Data Extraction and Analysis

Health strategy documents were reviewed manually by the research team. A structured comparative document review approach was used to identify and synthesize recurring policy priorities across the selected countries. Data extraction was conducted following the mentioned analytical framework. The review focused on identifying how public health priorities were represented within national policy documents rather than conducting formal qualitative coding or quantitative text analysis. Information extracted from each document included demographic and health indicators, leading causes of mortality, policy priorities, system challenges, and references to social and environmental determinants of health.

To facilitate regional comparison, extracted information was organized into standardized comparative tables. Two reviewers independently examined documents from their assigned region using the standardized extraction framework (Appendix A). For each document, all information relevant to the five predefined analytical domains was extracted and organized into standardized comparative tables. Uncertainties regarding data extraction or interpretation were resolved through discussion and consensus. The resulting datasets were subsequently reviewed jointly by the research team to identify recurrent policy patterns, similarities, and region-specific differences.

### 2.5. Ethical Considerations

This review used only publicly accessible documents and did not involve human subjects; therefore, ethical approval was not required.

## 3. Results

A total of 64 national health strategy documents were included in the review, comprising 12 documents from Latin America and 52 from Africa. In countries where multiple strategic documents were available, the most recent comprehensive national health strategy was selected according to the predefined inclusion criteria (Figure 2). The analysis of national health strategies and policy documents highlights distinct yet overlapping health priorities across selected countries in Latin America and Africa, categorized by five key public health domains: social determinants, environmental health, infectious diseases, non-communicable diseases (NCDs), and immunization. Table 1 and Table 2 summarize recurring policy priorities and health system challenges identified across the reviewed policy documents in selected Latin American and African countries, respectively. While Table 1 and Table 2 summarize the recurring policy priorities identified across the reviewed documents, the narrative synthesis presented below focuses on broader cross-regional patterns, contrasting policy emphases, and interpretive insights that emerge from the comparative analysis.

Policy documents from the reviewed Latin American countries tend to frame health challenges in relation to health system fragmentation, migration pressures, epidemiological transition, and continuity of chronic disease management. In contrast, policy documents from the reviewed African countries try to emphasize foundational access to health care services, communicable disease burdens, infrastructure limitations, and vulnerabilities associated with under-resourced health systems.

At the same time, selected countries from both regions show attention to non-communicable diseases, environmental risks, and health system resilience, suggesting areas of convergence in public health prioritization despite differing institutional and socioeconomic contexts.

### 3.1. Social Determinants of Health

Social determinants of health emerged as a recurrent priority across the reviewed national policy documents, although the specific issues emphasized varied between regions. In the selected Latin American countries, migration-related health challenges were explicitly addressed in several national strategies, particularly in Argentina, Colombia, and Peru. Common priorities included reducing inequities in access to health care, addressing high out-of-pocket expenditures, and improving access to services for socially vulnerable and marginalized populations. Several policy documents also highlighted the need to strengthen social protection mechanisms and address the health impacts of migration, gender-based violence, and food insecurity to promote more equitable access to care.

In the selected African countries, policy documents primarily emphasized structural social vulnerabilities, including poverty, unemployment, food insecurity, and limited access to education and health services. Across many of the reviewed national strategies, these challenges were discussed alongside deficiencies in water, sanitation, and hygiene (WASH) services, inadequate social protection systems, and persistent gender inequalities. Several countries also identified governance challenges and socioeconomic inequities as important barriers to improving population health and achieving equitable access to health services.

### 3.2. Environmental Health

Environmental health emerged as a recurrent priority across the reviewed national policy documents, although the specific environmental challenges and policy responses varied by regional and national context. In several Latin American countries, policy documents emphasized environmental concerns related to deforestation, air and water pollution, mining activities, and climate-sensitive risks. Some national strategies also highlighted increasing exposure to wildfires and their effects on air quality, while others identified weaknesses in environmental governance and waste management systems as barriers to protecting population health.

In the reviewed African policy documents, environmental health priorities primarily focused on the health impacts of rapid urbanization, inadequate sanitation, limited waste management infrastructure, and vulnerability to climate-related hazards such as droughts, floods, and heatwaves. Several policy documents identified environmental determinants as important contributors to population health risks, although the specific environmental exposures and associated health outcomes varied across countries. Commonly described challenges included limited access to safe water and sanitation, poor waste management, and climate-related environmental hazards that increase the burden of communicable diseases and, in some settings, contribute to non-communicable health risks.

Across both regions, additional environmental priorities included exposure to industrial chemicals, agricultural pesticide use, mining-related contamination, and food safety risks. However, the emphasis placed on these issues, as well as the level of detail provided, differed considerably among the reviewed national policy documents.

### 3.3. Infectious Diseases

In the reviewed Latin American policy documents, infectious diseases such as dengue, tuberculosis, malaria, Chagas disease, yellow fever, and sexually transmitted infections (STIs), including HIV, were identified as ongoing public health concerns. These challenges are primarily associated with barriers to prevention, timely diagnosis, continuity of care, and access to health services among vulnerable and underserved populations. Gaps in surveillance, diagnostic capacity, and treatment services further complicate disease control and management. In the reviewed African countries, the infectious disease burden remains particularly acute, with high incidence and mortality associated with HIV, malaria, and tuberculosis. Limited access to health care facilities, especially in rural areas, and underdeveloped surveillance and response systems contribute to ongoing challenges in disease prevention, control, and treatment.

### 3.4. Non-Communicable Diseases

Non-communicable diseases (NCDs) are a growing concern in both regions, reflecting an ongoing epidemiological transition. In the selected countries from Latin America, lifestyle-related illnesses such as obesity, diabetes, cancer, and cardiovascular diseases are increasingly common and represent a leading cause of disability-adjusted life years (DALYs). While some infrastructure for diagnosis and treatment exists, it remains unevenly distributed. In the reviewed African countries, policy documents tend to emphasize similar trends but with more pronounced barriers due to weak health care infrastructure, limited diagnostic tools, and scarce availability of chronic disease medications. Several policy documents identify limitations in screening and early intervention programs, which hinder timely management.

### 3.5. Immunization

In the reviewed Latin American policy documents, immunization systems are primarily described as relatively well-established, supported by long-standing public health programs and coordinated procurement mechanisms. However, several documents also highlight disruptions associated with the COVID-19 pandemic, including challenges in maintaining coverage levels, logistical constraints, and pressures on financing and service continuity in some countries. Although the proportion of zero-dose children remains substantially lower than in most selected African countries, policy documents increasingly recognize the need to recover missed vaccinations and reach underserved populations affected by geographic, social, and economic barriers.

In the reviewed African policy documents, immunization is consistently identified as a priority area, with particular emphasis on gaps in coverage among under- and unvaccinated populations, especially in remote and underserved settings. Commonly reported challenges include limitations in supply chains, cold-chain capacity, vaccine delivery infrastructure, and domestic production capacity. These constraints are primarily linked within policy documents to vulnerabilities associated with vaccine-preventable disease outbreaks and unequal access to routine immunization services. Several policy documents also highlight the persistence of large zero-dose populations, which remain a major challenge to achieving equitable immunization coverage.

Overall, the reviewed policy documents suggest that while immunization remains a central component of public health strategies in both regions, the framing of challenges differs across contexts. Latin American documents try to emphasize system resilience, recovery of missed vaccinations, and reaching pockets of under-immunized populations, whereas included African documents place greater emphasis on expanding foundational access, reducing the number of zero-dose children, strengthening delivery systems, and addressing structural gaps in coverage.

## 4. Discussion

The comparative analysis suggests areas of convergence and divergence in how public health priorities are framed in national policy documents across the selected countries from both regions. Despite differences in historical, political, and socioeconomic contexts, both Latin America and Africa face layered health system challenges spanning social, environmental, and epidemiological domains [19]. The findings indicate that while several priorities are shared, many are shaped by region-specific conditions, underscoring the need for context-sensitive approaches to health system planning and policy development.

The comparative approach adopted in this study is not intended to imply that Latin America and Africa are homogeneous regions or directly comparable in all respects. Rather, these regions were selected because they face several shared public health challenges, including the dual burden of communicable and non-communicable diseases, persistent health inequities, environmental health threats, and the need to strengthen health systems under resource constraints [20,21,22]. At the same time, important differences in demographic, socioeconomic, epidemiological, and health system contexts provide an opportunity to examine how similar challenges are prioritized within different policy environments [22]. This perspective supports South–South learning by identifying common priorities, complementary policy approaches, and context-specific strategies that may inform future policy development while recognizing that national health strategies reflect policy priorities rather than implementation capacity, resource allocation, or health system performance [23]. Consequently, the findings should be interpreted as comparative insights into policy framing across diverse national contexts rather than as evidence of policy effectiveness or implementation success.

### 4.1. Social Determinants of Health

Our findings indicate that social determinants of health were consistently recognized as major policy priorities across the reviewed national health strategies, although the specific determinants and vulnerable populations emphasized varied between Latin America and Africa. Across both regions, policy documents highlighted persistent inequities in access to health services associated with socioeconomic status, geography, education, gender, ethnicity, and migration status. These structural factors were primarily described as contributing to unequal access to preventive and curative services, particularly among rural populations, informal workers, migrants, and Indigenous communities [24,25,26].

The reviewed policy documents also emphasized that these inequities are closely linked to broader health system challenges. Limited health care infrastructure, shortages of trained health professionals, fragmented governance, and insufficient financing were primarily identified as barriers to equitable service delivery. Several national strategies further highlighted underinvestment in primary health care and weaknesses in health system organization as constraints to achieving Universal Health Coverage [27]. These findings reinforce the importance of addressing social determinants alongside health system strengthening rather than through isolated disease-specific interventions.

Although social determinants were prioritized across both regions, the way they were framed differed according to regional contexts. In the reviewed African policy documents, structural vulnerabilities were primarily described in relation to poverty, rural isolation, food insecurity, unemployment, and gender inequalities affecting access to health services, particularly for women and children [19]. In contrast, Latin American policy documents tend to emphasize migration, urban–rural disparities, informal employment, and the social exclusion of Indigenous and other marginalized populations as key determinants of health inequities [10,25]. These differences suggest that while countries recognize similar overarching goals of reducing health inequities, national strategies adapt these priorities to their specific demographic, socioeconomic, and institutional contexts.

These findings are consistent with the WHO Commission on Social Determinants of Health and subsequent evidence demonstrating that reducing health inequities requires coordinated action across sectors, including education, employment, housing, social protection, and health care [24,25]. Previous studies have similarly emphasized that social determinants remain fundamental drivers of health outcomes and access to care [28]. Our comparative review extends this evidence by demonstrating how these priorities are operationalized within national health strategies and by highlighting important differences among the reviewed documents, structural vulnerabilities, and policy responses emphasized across Latin America and Africa. These findings underscore the importance of designing context-specific, equity-oriented public health policies rather than applying uniform approaches across diverse health system settings.

### 4.2. Environmental Health

Our findings indicate that environmental health is recognized as an important policy priority across the reviewed national health strategies, although the environmental risks emphasized vary according to regional and national contexts. In the reviewed Latin American countries, policy documents primarily focused on environmental challenges related to deforestation, air and water pollution, extractive industries, and land-use changes, reflecting the region’s increasing concern with environmental degradation and its implications for population health [29]. In contrast, the reviewed African policy documents tend to emphasize challenges associated with rapid urbanization, inadequate sanitation, limited waste management infrastructure, and climate-related hazards such as droughts, floods, and extreme heat, highlighting the continued importance of basic environmental health services and climate resilience [30].

Across both regions, the reviewed policy documents identified environmental determinants as important contributors to population health risks, although the specific environmental exposures and associated health outcomes differed across countries. Several national strategies described limited access to safe water and sanitation, poor waste management, and climate-related hazards as factors contributing to communicable disease transmission, while others also recognized the growing contribution of air pollution, environmental degradation, and chemical exposures to non-communicable diseases. These findings suggest that environmental health is increasingly viewed within national strategies as an integral component of broader public health planning rather than as an isolated environmental concern [28].

Our findings are consistent with growing international evidence recognizing environmental determinants as fundamental drivers of population health and health inequities. The World Health Organization and the Lancet Countdown on Health and Climate Change have emphasized that climate change, environmental degradation, unsafe water, inadequate sanitation, and air pollution are increasingly influencing disease patterns while placing additional pressure on health systems [31,32,33]. Similarly, recent studies have highlighted the importance of developing climate-resilient health systems capable of responding to increasingly frequent environmental disruptions [34,35,36]. The findings of our comparative review demonstrate that these global priorities are reflected in national policy documents, although the environmental risks and policy responses emphasized differ according to local ecological, socioeconomic, and institutional contexts.

These differences observed among the reviewed documents likely reflect distinct environmental exposures, development trajectories, and institutional capacities. Nevertheless, the convergence observed across the reviewed policy documents underscores the growing recognition that environmental governance, climate adaptation, and health system strengthening are closely interconnected. Integrating environmental health into primary health care, multisectoral governance, and national public health planning may therefore be essential for reducing health inequities and improving health system resilience in both regions.

### 4.3. Infectious Diseases

Our findings indicate that infectious diseases remain central priorities within the reviewed national health strategies, although the specific diseases emphasized reflect the epidemiological profiles of each region. In the reviewed African countries, policy documents consistently prioritized HIV/AIDS, tuberculosis, and malaria, while also highlighting persistent challenges related to surveillance capacity, laboratory infrastructure, workforce shortages, and equitable access to diagnosis and treatment, particularly in rural and conflict-affected settings [37]. In contrast, policy documents from the reviewed Latin American countries tend to emphasize dengue, tuberculosis, malaria, Chagas disease, yellow fever, sexually transmitted infections, and HIV, reflecting the coexistence of endemic infectious diseases and emerging public health threats [10,25].

Across both regions, the reviewed policy documents emphasized that effective infectious disease control depends not only on disease-specific programs but also on broader health system capacity. Several national strategies identified weaknesses in surveillance systems, laboratory networks, workforce availability, and equitable access to preventive and curative services as persistent barriers to disease control. These findings suggest that strengthening health system functions, including surveillance, diagnostics, workforce capacity, and service delivery, remains a common strategic priority despite important regional differences in disease burden [38].

Our findings are consistent with previous evidence demonstrating that infectious disease priorities continue to be shaped by local epidemiological conditions while increasingly reflecting broader global health security and health system resilience agendas [39,40,41]. Following the COVID-19 pandemic, international organizations have emphasized the importance of strengthening integrated surveillance systems, laboratory capacity, emergency preparedness, and resilient health systems capable of responding to both endemic and emerging infectious diseases [42]. The reviewed policy documents reflect these global priorities while adapting them to country-specific epidemiological and institutional contexts.

Although the infectious disease profiles differed substantially between Latin America and Africa, both regions recognized that reducing the burden of infectious diseases requires coordinated investments in prevention, surveillance, early diagnosis, treatment, and equitable access to essential health services. These findings highlight the importance of strengthening primary health care and integrated public health systems as complementary strategies for improving preparedness, reducing health inequities, and enhancing resilience to future infectious disease threats [43,44,45].

Finally, these findings should be interpreted within the scope of this study. Our analysis examines how infectious disease priorities are articulated within national policy documents rather than evaluating epidemiological trends, program implementation, or health outcomes. Consequently, the discussion reflects the strategic priorities identified in national health policies and should not be interpreted as a direct assessment of disease burden or health system performance.

### 4.4. Non-Communicable Diseases

Our findings indicate that non-communicable diseases (NCDs) have become prominent priorities within the reviewed national health strategies across both Latin America and Africa, reflecting the ongoing epidemiological transition occurring in many low- and middle-income countries. Cardiovascular diseases, diabetes, cancer, chronic respiratory diseases, and obesity were consistently identified as growing public health concerns. However, the reviewed policy documents also highlighted persistent challenges related to prevention, early detection, long-term disease management, and equitable access to essential health services.

Although NCDs were prioritized across both regions, the barriers to effective implementation differed according to health system context. In the reviewed African countries, policy documents tend to emphasize limited diagnostic capacity, shortages of trained health professionals, inadequate access to essential medicines, and constrained health system resources as major obstacles to improving chronic disease care [19]. In contrast, policy documents from the reviewed Latin American countries primarily described challenges related to health system fragmentation, inequalities in access to specialized services, and insufficient coordination across levels of care. Despite these contextual differences, both regions identified weaknesses in continuity of care, community-based prevention, and long-term follow-up as important barriers to effective NCD management [46,47,48].

These findings are consistent with global evidence demonstrating that NCDs now account for the majority of premature mortality worldwide and represent one of the greatest challenges for contemporary health systems. The World Health Organization and Global Burden of Disease studies have consistently shown that population ageing, urbanization, lifestyle changes, and demographic transitions have substantially increased the burden of chronic diseases, particularly in low- and middle-income countries [8,48,49]. Our findings suggest that national policy documents increasingly recognize these epidemiological trends and have incorporated NCD prevention and control as central strategic priorities.

The reviewed policy documents also reinforce the growing international consensus that effective NCD control requires strengthening primary health care rather than relying primarily on disease-specific programs. Previous studies have demonstrated that comprehensive primary health care can improve prevention, facilitate early diagnosis, strengthen continuity of care, and reduce avoidable complications associated with chronic diseases [50,51,52]. Nevertheless, our findings indicate that many countries continue to face important implementation challenges in translating these strategic priorities into accessible, integrated, and person-centered health services.

Overall, the convergence observed across the reviewed national strategies suggests increasing recognition that addressing NCDs requires integrated health system approaches combining health promotion, disease prevention, equitable access to care, and long-term chronic disease management. While implementation priorities differ according to regional contexts and institutional capacity, strengthening primary health care, expanding preventive services, and improving continuity of care emerge as common policy priorities for reducing the growing burden of NCDs [53].

### 4.5. Immunization

Our findings indicate that immunization remains one of the most consistently prioritized public health interventions across the reviewed national health strategies, although the principal challenges and policy responses differ between Latin America and Africa. Across both regions, vaccination is recognized as an essential strategy for preventing infectious diseases, reducing child mortality, and strengthening health system resilience. However, the reviewed policy documents reveal important differences in how countries frame the barriers to achieving and sustaining high vaccination coverage.

In the reviewed Latin American countries, national strategies generally described immunization systems as relatively well established, supported by long-standing public health programs and regional procurement mechanisms such as the PAHO Revolving Fund [54]. Nevertheless, several policy documents acknowledged that the COVID-19 pandemic disrupted routine immunization services through challenges related to logistics, financing, and service delivery, creating coverage gaps among specific populations and reinforcing the need to strengthen recovery strategies and maintain resilient immunization programs [55,56].

In contrast, the reviewed African policy documents tend to emphasize the need to expand foundational immunization capacity. Common priorities included strengthening supply chains, improving cold-chain infrastructure, increasing domestic financing, expanding outreach services, and reaching zero-dose and under-immunized children, particularly in geographically remote and underserved communities [57]. Several strategies also highlighted the importance of developing local manufacturing capacity, strengthening regulatory systems, and improving vaccine delivery as part of broader health system strengthening efforts.

These findings are consistent with the goals of the WHO Immunization Agenda 2030, which emphasizes equitable access to vaccines, resilient immunization systems, and the reduction of zero-dose populations as global priorities [58]. Previous studies have similarly shown that sustaining high vaccination coverage depends not only on vaccine availability but also on strong primary health care systems, effective governance, reliable financing, resilient supply chains, and community trust [59]. Our comparative review demonstrates that these global priorities are reflected across both regions while being adapted to different epidemiological, institutional, and financing contexts.

The reviewed policy documents also illustrate the important influence of regional and international organizations in shaping immunization policies. In Latin America, PAHO has played a longstanding role in supporting vaccine procurement, technical cooperation, and regional coordination. In Africa, initiatives such as the African Medicines Agency, the African Medicines Regulatory Harmonization initiative, and procurement platforms coordinated through Africa CDC reflect increasing efforts to strengthen regulatory capacity, regional collaboration, and vaccine security [39,40,41]. Although these mechanisms differ in their maturity and institutional organization, they demonstrate growing regional commitment to strengthening immunization systems and improving equitable access to vaccines.

Overall, the convergence observed across the reviewed national strategies suggests increasing recognition that sustainable immunization programs depend on more than vaccine procurement alone. Strengthening primary health care, improving governance and financing, expanding surveillance and supply chain capacity, and addressing inequities in access remain essential components of resilient immunization systems. At the same time, the differences observed among the reviewed documents identified in this review highlight that effective immunization policies must be tailored to local health system capacities and implementation contexts rather than relying on uniform approaches.

### 4.6. Strengths and Limitations

This review has several strengths and limitations that should be considered when interpreting its findings. A key strength lies in its comparative and multi-domain analytical framework, which integrates evidence across infectious disease, NCD, vaccination, social, and environmental health domains. By synthesizing national policy documents alongside global data sources, this study provides a regionally grounded understanding of health system priorities and policy directions across Latin America and Africa. Moreover, its focus on policy-level analysis enables identification of structural and systemic trends that often transcend country borders.

However, several limitations must be acknowledged. First, the review’s reliance on national strategic and policy documents may not fully capture real-time population health needs or the lived experiences of marginalized and vulnerable groups. These sources often reflect official priorities rather than on-the-ground realities. Second, data availability and quality varied considerably between countries, and some indicators were inconsistently reported or not disaggregated by gender, age, or socioeconomic status, limiting the depth of comparative analysis.

Third, the scope of country coverage, twelve Latin American and 52 African countries, restricts the generalizability of findings. While these countries were chosen for data availability and regional diversity, they may not fully represent the heterogeneity of health systems and environmental contexts across both continents.

Finally, this study analyzes policy priorities as reflected in national documents and does not directly measure epidemiological outcomes or population-level health indicators. The analysis does not attempt to measure social inequality directly. Rather, it examines how inequality, vulnerability, and inequitable access are represented within national policy documents and supporting data sources. The limited availability of disaggregated indicators in some documents constrained the depth of comparison across gender, age, territory, and socioeconomic status.

An important consideration in interpreting these findings is that national policy documents are inherently normative and strategic texts. They reflect not only epidemiological concerns and health system priorities, but also institutional expectations, international reporting frameworks, donor agendas, political considerations, and prevailing global health discourse. Consequently, similarities observed across policy documents may partially reflect broader processes of policy diffusion, institutional alignment, and shared global health priorities rather than identical on-the-ground conditions.

The findings should therefore be interpreted as comparative insights into policy framing and strategic prioritization rather than direct measurement of health system performance or population health outcomes. This dynamic is particularly relevant in domains such as non-communicable diseases, immunization, and environmental health, where international organizations and financing mechanisms frequently shape national strategic language and reporting priorities. Similarities in policy framing across countries may therefore reflect shared participation in global health governance processes alongside genuine convergence in public health concerns.

Across both regions, many of the challenges identified in the reviewed policy documents, including unequal access to care, environmental vulnerabilities, chronic disease burdens, and gaps in preventive services, highlight the central role of Primary Health Care (PHC) in advancing Universal Health Coverage (UHC). PHC-oriented approaches may help strengthen continuity of care, coordination across services, community-based prevention, and equitable access to essential health interventions, particularly among socially vulnerable and underserved populations [60].

The findings also suggest that PHC systems may play an increasingly important role in responding to climate-sensitive health risks and the growing burden of non-communicable diseases. Integrating environmental health, immunization, and social determinants of health within PHC-oriented strategies may support more resilient and context-sensitive pathways toward UHC implementation across diverse health system settings [61].

Beyond its role in service delivery, recent PHC reforms demonstrate how primary care can operationalize responses to complex public health challenges. For example, Chile has advanced a people-centered PHC model that integrates health promotion, chronic disease prevention, mental health, community participation, and intersectoral action within local health networks [62]. Similar experiences across Latin America have strengthened community-based prevention, expanded outreach to vulnerable populations, and improved continuity of care through multidisciplinary PHC teams [60]. In African settings from selected countries, community health worker programs have played a central role in extending preventive services, supporting immunization campaigns, strengthening disease surveillance, and enhancing resilience during public health emergencies [63]. Together, these experiences illustrate that PHC can serve as a platform not only for achieving Universal Health Coverage, but also for addressing environmental health risks, reducing health inequities, and strengthening community resilience through multisectoral collaboration.

## 5. Conclusions

This study provides a comparative analysis of how public health priorities are articulated in national policy documents across selected countries in Latin America and Africa. By examining five key domains, social determinants of health, environmental health, infectious diseases, non-communicable diseases, and immunization, the findings highlight both shared and context-specific policy concerns across the reviewed country sample.

Across the reviewed Latin American countries, policy documents tend to identify health system fragmentation, migration-related pressures, social exclusion, and increasing burdens of non-communicable diseases as key challenges. In the reviewed African countries, policy priorities tend to emphasize health system resource constraints, limited access to preventive services, persistent communicable disease burdens, and climate-related vulnerabilities. These patterns should not be interpreted as uniform characteristics of entire regions, but rather as tendencies observed within the included policy documents.

While both regions share concerns related to rising NCDs and environmental risks, the framing of these challenges within policy documents reflects differences in health system capacity, financing structures, governance arrangements, and stages of epidemiological transition. These findings underscore the importance of context-sensitive approaches to health system strengthening rather than one-size-fits-all solutions.

The comparative findings also suggest several opportunities for cross-regional learning and policy adaptation. Community health worker models described in the reviewed policy documents may represent opportunities for future cross-regional learning, although their applicability should be evaluated within the context of individual health systems. Conversely, Regional procurement and immunization coordination mechanisms identified in this review may serve as examples for future comparative policy research, rather than directly transferable policy models.

Similarly, both regions may benefit from greater collaboration in areas such as climate-resilient health systems, primary health care strengthening, local pharmaceutical production capacity, and strategies addressing the social determinants of health. Strengthening PHC systems may be particularly important for improving continuity of care, expanding preventive services, supporting immunization delivery, and advancing more equitable pathways toward Universal Health Coverage. These areas represent potential pathways for South–South cooperation and context-sensitive policy innovation.

Finally, this study demonstrates the value of comparative policy analysis for identifying convergences and divergences in how countries define and prioritize public health challenges. Such comparative approaches can support more context-sensitive policy development by identifying both shared vulnerabilities and region-specific institutional responses. Rather than promoting uniform policy models, the findings highlight the importance of adaptive strategies that consider differences in health system capacity, governance structures, environmental risks, and social conditions across countries.

Future research should further integrate comparative policy analysis with epidemiological and implementation data to better understand how national policy priorities translate into health system performance and population health outcomes.

## Figures and Tables

**Table 1:** Recurring policy priorities identified in reviewed Latin American policy documents.

Health Domain	Key Challenges	Contextual Indicators
Social Determinants of Health	Low investment in health, migration stress, inequities in access, gender-based violence, out-of-pocket expenses.	Health care access gaps, social protection coverage, migration rates, gender-based violence rates.
Environmental Health Priorities	Air and water pollution, deforestation, mining impacts, weak environmental health policy.	Deforestation rate, urban air pollution levels, water contamination data, climate policy coverage.
Infectious Diseases	HIV, STIs, vector-borne diseases; gaps in access to diagnostics and care in marginalized populations.	Prevalence of HIV/STIs, incidence of dengue/chagas, access to infectious disease clinics.
Non-Communicable Diseases (NCDs)	Obesity, diabetes, heart disease rising; lack of integrated chronic disease management.	NCD mortality rates, obesity prevalence, physical inactivity levels, screening program access.
Immunization and Vaccine Priorities	Weakened immunization programs, centralized procurement challenges, COVID-19 vaccine hesitancy.	Routine immunization rates, COVID-19 vaccination coverage, vaccine purchase centralization.

**Table 2:** Recurring policy priorities identified in reviewed African policy documents.

Health Domain	Key Challenges	Contextual Indicators
Social Determinants of Health	Social inequalities, unemployment, food insecurity, limited access to health and education.	Education levels, unemployment rate, WASH access, social health protection levels.
Environmental Health Priorities	Poor sanitation, waste mismanagement, high vulnerability to climate impacts, waterborne diseases	Air and water quality indices, climate vulnerability maps, access to safe drinking water.
Infectious Diseases	High burden of HIV, TB, malaria; limited surveillance, diagnostics, treatment in rural areas.	HIV/TB/malaria incidence and mortality, testing coverage, rural health care facility density.
Non-Communicable Diseases (NCDs)	Growing burden of NCDs, weak primary care systems, limited diagnostics and drug access.	NCD share of total mortality, diagnostic infrastructure availability, health workforce capacity.
Immunization and Vaccine Priorities	Large zero-dose population, limited vaccine supply and delivery infrastructure.	Zero-dose ^1^ children’s statistics, cold chain availability, national immunization coverage.

^1^ Zero-dose definition: refers to children who have not received any routine childhood immunizations. The first dose of diphtheria-tetanus-pertussis vaccine (DTP-1) is the zero-dose reference indicator for this guidance [18].

## Data Availability

The data supporting the findings of this study are derived entirely from publicly available sources. National health strategy and policy documents analyzed in this review can be accessed through the official websites of the respective Ministries of Health in Latin American and African countries. Additional datasets and global health indicators were obtained from publicly accessible repositories maintained by the World Health Organization (WHO) and the Pan American Health Organization (PAHO). Specific URLs, report titles, and access dates for all referenced databases, and policy documents were verified and updated in the reference list to improve reproducibility and transparency.

## Abbreviations

The following abbreviations are used in this manuscript:

**Abbreviation**	**Meaning**
DALY	Disability-Adjusted Life Year
ECLAC	Economic Commission for Latin America and the Caribbean
GAVI	Global Alliance for Vaccines and Immunization
HIV	Human Immunodeficiency Virus
NCD	Non-Communicable Disease
PAHO	Pan American Health Organization
SDG	Sustainable Development Goal
STI	Sexually Transmitted Infection
TB	Tuberculosis
UHC	Universal Health Coverage
UNDP	United Nations Development Programme
UNHCR	United Nations High Commissioner for Refugees
UNICEF	United Nations Children’s Fund
WASH	Water, Sanitation, and Hygiene
WHO	World Health Organization

## Appendix A

The table in Appendix A provides an example of the structured extraction framework used during the comparative review of national health strategy and policy documents. Information was manually extracted and organized across five predefined analytical domains to facilitate comparative synthesis across the reviewed countries.

**Table A1:** Standardized Comparative Extraction Framework.

Region	Country	Population/Demographic Indicators	Health System Context	Social Determinants of Health	Environmental Health	Infectious Diseases	Immunization/Vaccines	Non-Communicable Diseases	Key Policy Priorities Identified	Contextual Notes	Main Policy Document/Source
Latin America	Colombia	Population size, life expectancy, mortality indicators	Fragmented mixed health system	Migration, inequities in access, social exclusion	Air pollution, mining-related exposure	Vector-borne diseases, STIs	Post-COVID recovery, coverage gaps	Obesity, diabetes	PHC strengthening, health equity	Migration-related service pressures	National Health Plan
Latin America	Peru	Population size, maternal mortality	Decentralized public system	Rural inequities, Indigenous populations	Deforestation, water quality	Dengue, TB	Immunization recovery strategies	Chronic disease prevention	Environmental governance, PHC	Amazon region vulnerability	Ministry of Health Strategy
Africa	Kenya	Population size, U5 mortality	Resource-constrained health system	Poverty, WASH limitations	Drought, sanitation	HIV, TB, malaria	Zero-dose populations	Increasing NCD burden	Health access expansion	Rural access barriers	National Health Sector Plan
Africa	Ghana	Life expectancy, MMR	Mixed public health financing	Food insecurity, unemployment	Flood vulnerability	Malaria	Cold-chain strengthening	Hypertension, diabetes	Community health worker expansion	Climate-sensitive risks	National Health Policy

Note: The extraction framework was used to organize and synthesize information from publicly available national health strategies and policy documents across the five predefined analytical domains. The framework supported comparative identification of recurring policy priorities, contextual challenges, and health system characteristics across the reviewed countries.
